# Characterization of Site-Specific Phosphorylation of NF-*κ*B p65 in Retinal Cells in Response to High Glucose and Cytokine Polarization

**DOI:** 10.1155/2018/3020675

**Published:** 2018-04-26

**Authors:** Haoshen Shi, Elizabeth A. Berger

**Affiliations:** ^1^Department of Anatomy & Cell Biology, Wayne State University School of Medicine, Detroit, MI 48201, USA; ^2^Department of Ophthalmology, Kresge Eye Institute, Detroit, MI 48201, USA

## Abstract

**Background:**

Inflammation is an important contributor to the pathogenesis of diabetic retinopathy (DR). NF-*κ*B is a master transcriptional regulator for numerous inflammatory genes. Although NF-*κ*B is comprised of multiple subunits, p65 has received the most attention. However, the p65 subunit can be phosphorylated at numerous sites, for which the effects of DR-related conditions are not well characterized. Since dysregulation of NF-*κ*B has been linked to chronic inflammation, the current study examines site-specific p65 phosphorylation in retinal cells exposed to high glucose and investigates the effects of cytokine polarization.

**Methods:**

Phosphorylation of NF-*κ*B p65 sites was examined in human primary retinal endothelial cells (HREC) and MIO-M1 Müller cells after exposure to high glucose (HG) and pro- or anti-inflammatory cytokines. Related downstream gene activation was selectively measured by real-time RT-PCR, ELISA, and/or Western blot.

**Results:**

HG exposure resulted in differential phosphorylation of p65 subunit sites between HREC and Müller cells. Proinflammatory cytokines further increased phosphorylation of these sites and additional sites that were not altered in HG. In contrast, IL-4 exhibited a suppressive effect on the phosphorylation of p65 sites in both cell types and promoted I*κ*B*α* expression. Downstream inflammatory mediators were increased in response to proinflammatory cytokine treatment versus HG exposure. IL-4 inhibited proinflammatory cytokines, while IL-10 was enhanced despite HG exposure.

**Conclusion:**

The current study is the first to characterize HG-induced NF-*κ*B p65 phosphorylation after cytokine polarization. By understanding NF-*κ*B phosphorylation and cytokine influence during hyperglycemic conditions, intervention points can be identified for early-stage treatment of DR.

## 1. Introduction

Diabetes mellitus (DM) is a chronic, metabolic disease resulting in high blood glucose levels in the body. Diabetic retinopathy (DR) is a visually debilitating eye complication of diabetes that is the leading cause of blindness among working age patients in the United States [1]. This disease exhibits several pathological events associated with the retina. The early stage of DR, with its featured complications of microaneurysms, hard exudates, and progressing hemorrhages, continues to lack effective treatments [2]. Although intensive efforts have been put into understanding pathogenic mechanisms of DR, remarkable intervention points for treatment against nonproliferative stages of DR have yet to be found. Current antivascular endothelial growth factor (VEGF) treatments risk deleterious side effects such as endophthalmitis, intraocular inflammation, and elevated intraocular pressure [3], with compromised efficacy around 50% and requires repetitive administration [4].

Inflammation was found to be associated with diabetes back in the 1960s [5]; since then, leukostasis and associated local inflammatory activities have been demonstrated to be key contributors for retinal nonperfusion, retinal ischemia, and resultant retinal vascular leakage during DR [6–9]. The retinal endothelium is part of the blood-retinal barrier which isolates the retina from toxins, microorganisms, and proinflammatory leukocytes [10]. Retinal endothelial cells (RECs) line the microvasculature with surrounding pericytes [11]. They are directly exposed to hyperglycemic conditions and interact with infiltrating leukocytes. During inflammation, RECs respond to extracellular molecules secreted by both residential retinal cells as well as immune cells. While DR was traditionally characterized as a microvascular disease, it is now being viewed as a neural degenerative disease [12], as well. Müller cells are supporting glia in the retina and one of the earliest residential cellular responders during the pathogenesis of DR [13]. They actively react to inflammatory cytokines and secrete various molecules to modulate the microenvironment during the development of DR, including RECs [14]. Additionally, it has been demonstrated that reactive oxygen species (ROS) toxicity in RECs was derived from retinal Müller cells and pigment epithelial cells through paracrine effects rather than a direct effect of high glucose [15], suggesting a role for this cell type during the development of this disease.

NF-*κ*B is a major transcription factor evoked by a number of stimuli, including proinflammatory cytokines. Activation of NF-*κ*B further enhances the inflammatory response by inducing the transcription of a wide spectrum of inflammatory mediators related to leukocyte recruitment and cytokine production [16]. Five NF-*κ*B subunits have been well characterized and associated with its activity, including RelA (p65), RelB, p50, p52, and c-rel [17]. Under normal conditions, NF-*κ*B is sequestered within the cytoplasm through the direct interaction with inhibitor proteins such as I*κ*B*α*. Upon activation, the IKK complex phosphorylates I*κ*B*α*. Further ubiquitination and degradation of I*κ*B*α* releases the transcription-activating subunits of NF-*κ*B [17]. The classic pathway of NF-*κ*B activation is triggered by the IL-1 receptor (IL-1R), the TNF receptor (TNFR), and pattern recognition receptors (PRRs), through downstream activation of IKK*β* and I*κ*B*α* and release of p65-p50 [17]. In contrast, the noncanonical NF-*κ*B pathway is activated by CD40 ligand and lymphotoxin-*β* and depends on the activation of IKK*α* and release of p52-RelB [17]. When released, p65-p50 and RelB-p52 heterodimers translocate into the nucleus, recruiting transcription coactivators such as cAMP-response element binding protein (CREB) binding protein (CBP)/p300 and histone acetyltransferase (HAT), and activate transcription of downstream molecules by binding to target DNA elements [17].

Site-specific phosphorylation of the NF-*κ*B p65 subunit leads to the selective transcription of downstream proinflammatory genes [18]. As the best characterized subunit of NF-*κ*B, p65 and its phosphorylation is a pivotal point for canonical NF-*κ*B activation. However, multiple phosphorylation sites have been mapped in both the N-terminal Rel homology domain and the C-terminal transactivation domain of p65; Ser-205, Thr-254, Ser-276, Ser-281, and Ser-311 are located in the N-terminal Rel homology domain, while Thr-435, Ser-468, Thr-505, Ser-529, Ser-535, Ser-536, and Ser-547 are found in the C-terminal transactivation domain [18]. It has been shown that during inflammation, phosphorylation of Ser-276, Ser-281, Ser-311, Ser-468, Ser-529, Ser-536, and Thr-435 stimulates transcriptional activity, while Thr-254 is involved in stabilization and nuclear translocation [19]. Since the high glucose-induced influence on different NF-*κ*B p65 phosphorylation sites is unknown, we first sought to characterize these sites in RECs and Müller cells under such conditions.

Inflammation is a protective immunomodulatory response, during which proinflammatory mediators are counterbalanced by anti-inflammatory agents. During DR, proinflammatory cytokines, mainly interleukin-1 beta (IL-1*β*) and tumor necrosis factor-alpha (TNF-*α*), are produced by residential retinal cells, neutrophils, and macrophages and shape the inflammatory response that contributes to disease pathogenesis [20, 21]. Both of these inflammatory mediators are canonical NF-*κ*B pathway activators. In type 1 diabetes, IL-1*β* activates NF-*κ*B and induces pancreatic beta cell dysfunction and death [22], while in type 2 diabetes NF-*κ*B is constitutively activated by a low-grade, chronic state of inflammation [23]. Regarding DR, NF-*κ*B has been shown to be activated early and remain activated for up to 14 months in experimental animal models and cultured retinal cells [20]. In contrast, interleukin-4 (IL-4) is associated with anti-inflammatory immune responses and influences further differentiation of T cells into Th2 cells. It has potent anti-inflammatory effects on other leukocytes such as polymorphonuclear leukocytes (PMN) and monocytes [24]. It has been reported that IL-4 inhibits insulitis and diabetic mellitus by stimulating a Th2 response [25]. In terms of DR, clinical studies have indicated significantly elevated levels of IL-4 in both vitreous and aqueous humor in patients, together with other proinflammatory cytokines [26, 27]. However, little is known on the anti-inflammatory effect of IL-4 in DR. In addition, whether IL-4 has any regulatory effect on NF-*κ*B phosphorylation has yet to be found. As such, we sought to investigate the effects of cytokine polarization regarding differential site-specific phosphorylation of NF-*κ*B p65 in HREC and Müller cells exposed to high glucose.

## 2. Materials and Methods

### 2.1. Cell Culture and Cytokine Time Course Treatment

Primary HRECs (Cell Systems Corporation; Kirkland, WA) were grown in HREC medium containing microvascular growth supplements (MVGS; Invitrogen, Carlsbad, CA), 10 mg/mL gentamicin, and 0.25 mg/mL amphotericin B. All cells were used within six passages. The MIO-M1 Müller cell line was obtained from the UCL Institute of Ophthalmology, London, UK. MIO-M1 Müller cells were cultured in Dulbecco's modified Eagle medium (DMEM) with 10% fetal bovine serum (FBS; Invitrogen, Carlsbad, CA), 10 mg/mL gentamicin, and 0.25 mg/mL amphotericin B. Prior to experimentation, cells were transferred for four days to a high-glucose (25 mM) or normal-glucose (5 mM) medium (HREC medium or DMEM medium supplemented with glucose) with MVGS or FBS and antibiotics. Cells were then quiesced by removing MVGS or FBS for 24 h. Cells were treated with proinflammatory cytokine IL-1*β* (10 ng/mL, R&D Systems, Minneapolis, MN) or TNF-*α* (10 ng/mL, R&D Systems, Minneapolis, MN) versus anti-inflammatory cytokine IL-4 (20 ng/mL, R&D Systems, Minneapolis, MN) for 10 min (MIO-M1 only), 30 min, 2 h, and 24 h, followed by rinsing with cold PBS. Since Müller cells are early responders in DR and have similar characteristics with macrophages [28], an earlier time point of 10 minutes was added for the analysis of these cells. Cell collection was carried out as detailed below.

Previously, high osmolar conditions have been included as an additional control to determine if the observed *in vitro* effects were a result of either high-glucose treatment or increased osmolarity of the treatment media [29, 30]. Since it has been established that no differences were observed between high osmolarity and normal glucose, results were not included in the current study.

### 2.2. Western Blotting

Cells were collected in lysis buffer containing protease and phosphatase inhibitors for protein isolation. Cellular extracts were then prepared by sonication, and total protein concentration was determined for Western blot analyses. Proteins were separated on 4–20% Tris-glycine gels (Invitrogen, Carlsbad, CA) and transferred to nitrocellulose membranes. After blocking membranes in TBST (10 mM Tris-HCl buffer, pH 8.0, 150 mM NaCl, and 0.1% Tween 20) and 5% (*w*/*v*) BSA at r.t. for 60 min, membranes were incubated overnight at 4°C with antigen-specific primary antibodies. The primary antibodies were used as follows: anti-NF-*κ*B p65 (phospho Thr-254), anti-NF-*κ*B p65 (phospho Ser-276), anti-NF-*κ*B p65 (phospho Ser-281), anti-NF-*κ*B p65 (phosphor Ser-311), anti-NF-*κ*B p65 (phospho Ser-468), anti-NF-*κ*B p65 (phospho Ser-529), anti-NF-*κ*B p65 (phospho Thr-435), and anti-I*κ*B*α* (Abcam, San Francisco, CA); anti-NF-*κ*B p65 (phospho Ser-536) and antivascular cell adhesion protein 1 (VCAM-1) (Cell Signaling Technology, Danvers, MA); and anti-COX-2 and anti-*β*-actin (Santa Cruz, Santa Cruz, CA). Blots were then incubated with species-specific HRP-conjugated secondary antibodies for 2 h at r.t. Proteins were visualized by incubation with a chemiluminescence substrate kit (Thermo Fisher Scientific, Waltham, MA). Western blot images were collected (Azure Biosystem C500, Dublin, CA), and target protein expression was quantified (Image Studio Lite software) after normalizing to *β*-actin. One representative blot is shown.

### 2.3. Real-Time RT-PCR

RNA was extracted by RNA STAT-60 (Tel-Test, Friendswood, TX, USA) per the manufacturer's protocol and subjected to real-time RT-PCR analyses. Total RNA extracted for HREC and MIO-M1 was quantitated by spectrophotometric determination (260 nm). Total RNA (100 ng) was reverse transcribed and used to produce a cDNA template as previously described [31]. cDNA products were diluted 1 : 20 with DEPC-treated water, and 2 *μ*L cDNA (10 *μ*L total reaction volume) was used for semiquantitative real-time RT-RT-PCR analysis (CFX Connect Real-Time RT-PCR Detection System; Bio-Rad, Hercules, CA, USA). All human primer pair sequences designed in the laboratory (PrimerQuest, Integrated DNA Technologies, Coralville, IA, USA) are listed in Table 1. RT-PCR amplification conditions were determined using routine methods [32]. Relative transcript levels were calculated using the relative standard curve method comparing the amount of targets normalized to an endogenous reference, *β*-actin. Data are shown as mean ± SD for relative transcript levels and represent at least two individual experiments.

### 2.4. ELISA

Intercellular adhesion molecule-1 (ICAM-1), interleukin-8 (IL-8), interleukin-10 (IL-10) (R&D Systems, Minneapolis, MN), IL-1*β*, and TNF-*α* (Thermo Fisher Scientific, Waltham, MA) ELISAs were used to measure protein expression in HREC and Müller cells. Cell lysates were collected and processed as described above. All samples were assayed in duplicate or triplicate per the manufacturer's instruction. Equal protein was loaded into all wells. The reported sensitivities of these assays are 0.254 ng/mL for ICAM-1, 7.5 pg/mL for IL-8, 3.9 pg/mL for IL-10, 1 pg/mL for IL-1*β*, and 1.7 pg/mL for TNF-*α*.

### 2.5. Statistical Analysis

All assays were carried out at least twice from three independent experiments, and the data are presented as mean ± SD. Data were analyzed by the analysis of variance (ANOVA) test following by Fisher's LSD test. *P* < 0.05 was considered to be statistically significant.

## 3. Results

### 3.1. I*κ*B*α* Levels Are Reduced in Response to IL-1*β*/TNF-*α*, Yet Restored with IL-4 after HG Exposure

**Figure 1:**
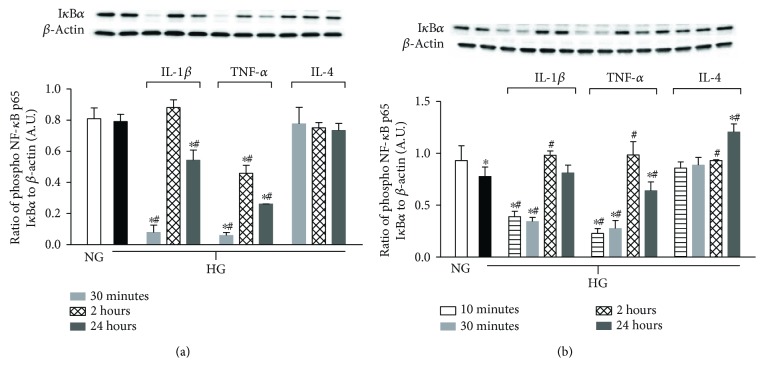
Degradation of I*κ*B*α* in HREC versus Müller cells when cultured in high glucose with cytokine treatments. HREC and Müller cells were cultured under normal-glucose (NG, 5 mM) and high-glucose (HG, 25 mM) conditions followed by TNF-*α* and IL-1*β* versus IL-4 treatment for 10 minutes (Müller cells only), 30 minutes, 2 hours, and 24 hours. Protein levels of I*κ*B*α* in HREC (a) and I*κ*B*α* in Müller cells (b) were detected by Western blot. Data shown are representative of 5 independent experiments in duplicate and are expressed as mean ± SD. ^∗^*P* < 0.05 versus NG and ^#^*P* < 0.05 versus HG.

Levels of I*κ*B*α*, a regulatory protein that inhibits NF-*κ*B, were assessed in HREC and Müller cells after high glucose exposure and cytokine treatment over time (Figure 1). Regarding HREC, there was no difference in I*κ*B*α* levels between normal and high glucose. However, in the presence of proinflammatory cytokines, IL-1*β* and TNF-*α*, I*κ*B*α* was significantly downregulated early (30 minutes). These levels increased at 2 h, but remained significantly reduced over NG and HG treatment groups at 24 h. In contrast, IL-4 treatment maintained I*κ*B*α* levels similar to controls.

In contrast to HREC, HG reduced I*κ*B*α* levels in Müller cells. However, similar trends were observed after cytokine treatments, where proinflammatory IL-1*β* and TNF-*α* significantly reduced I*κ*B*α* at early time points, which then appeared to peak at 2 h and decrease at 24 h (significant for TNF-*α* only). IL-4 treatment restored HG-induced downregulation of I*κ*B*α*, which was significantly higher than HG at 2 h and both NG and HG at 24 h.

### 3.2. Differential Phosphorylation of NF-*κ*B p65 Subunits When Exposed to High Glucose versus Inflammatory Cytokines in HREC and Müller Cells

**Figure 2:**
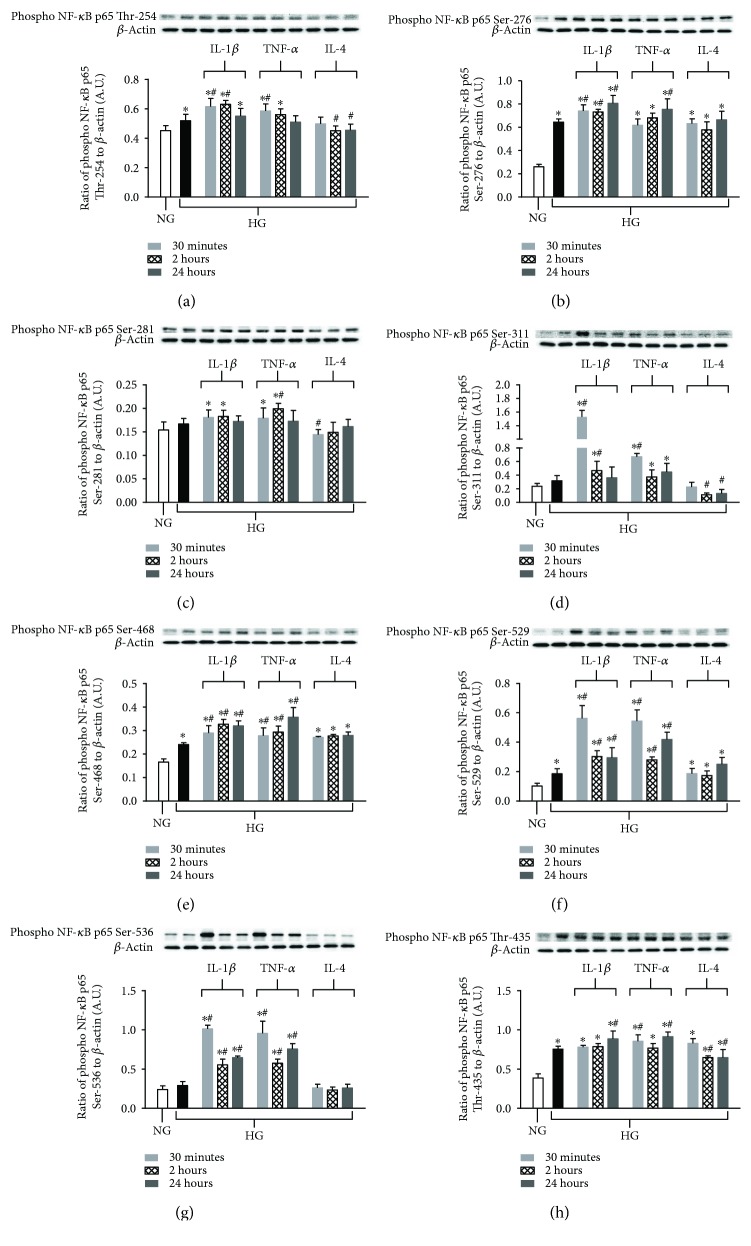
Differential site-specific phosphorylation of NF-*κ*B p65 after high glucose exposure and proinflammatory versus anti-inflammatory cytokine stimulation in HREC. HRECs were cultured under normal-glucose (NG, 5 mM) and high-glucose (HG, 25 mM) conditions followed by TNF-*α* and IL-1*β* versus IL-4 treatment for 30 minutes, 2 hours, and 24 hours. Protein levels of phosphorylated p65 Thr-254 (a), phosphorylated p65 Ser-276 (b), phosphorylated p65 Ser-281 (c), phosphorylated p65 Ser-311 (d), phosphorylated p65 Ser-468 (e), phosphorylated p65 Ser-529 (f), phosphorylated p65 Ser-536 (g), and phosphorylated p65 Thr-435 (h) were detected by Western blot. Data shown are representative of 5 independent experiments in duplicate and are expressed as mean ± SD. ^∗^*P* < 0.05 versus NG and ^#^*P* < 0.05 versus HG.

Next, we characterized the phosphorylation of NF-*κ*B p65 subunits in response to high glucose, proinflammatory NF-*κ*B activators (TNF-*α* and IL-1*β*), and anti-inflammatory IL-4 in HREC. As shown in Figure 2, high glucose upregulated phosphorylation at five out of the eight tested p65 sites, including Thr-254 (*A*), Ser-276 (*B*), Ser-468 (*E*), Ser-529 (*F*), and Thr-435 (*H*). No differences were observed for sites Ser-281 (*C*), Ser-311 (*D*), or Ser-536 (*G*) after exposure to high glucose only. When HRECs were exposed to high glucose in the presence of either IL-1*β* or TNF-*α*, all sites tested showed significantly increased phosphorylation compared to normal-glucose controls. Moreover, both of these cytokines further enhanced NF-*κ*B activation over high glucose-induced effects at most of the time points tested. Additionally, IL-1*β* appears to be a more potent stimulator of Ser-311 compared to TNF-*α* at 30 min after treatment. On the contrary, anti-inflammatory cytokine IL-4 suppressed high glucose-induced phosphorylation of p65 subunit sites Thr-254, Ser-281, and Thr-435. Although high glucose did not induce changes in Ser-281, Ser-311, or Ser-536, IL-4 treatment reduced the activation of NF-*κ*B at 30 minutes for Ser-281 and at 2 and 24 h for Ser-311 below basal levels observed with normal glucose. No effect was observed with Ser-536. There was no indication that IL-4 treatment increased phosphorylation beyond the observed high glucose-induced effects for sites Ser-276, Ser-468, and Ser-529.

**Figure 3:**
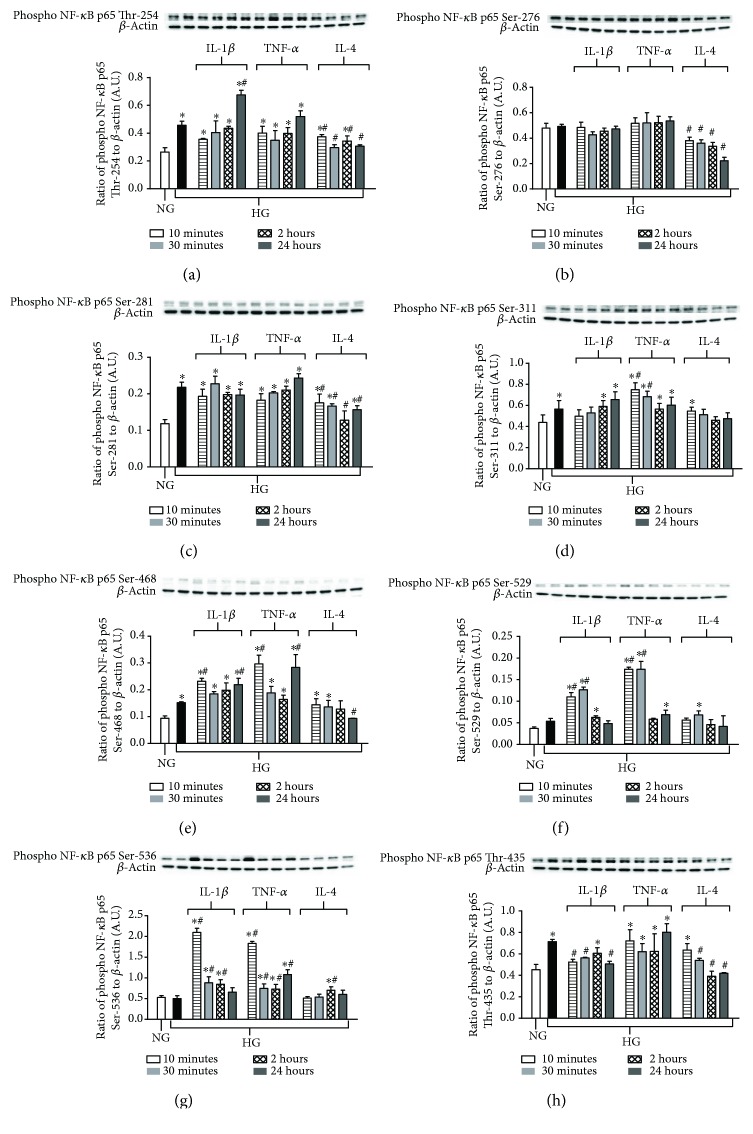
Site-specific differential phosphorylation of NF-*κ*B p65 in Müller cells after high glucose exposure and pro- versus anti-inflammatory cytokine stimulation. Müller cells were cultured under normal-glucose (NG, 5 mM) and high-glucose (HG, 25 mM) conditions followed by TNF-*α* and IL-1*β* versus IL-4 treatment for 10 minutes, 30 minutes, 2 hours, and 24 hours. Protein levels of phosphorylated p65 Thr-254 (a), phosphorylated p65 Ser-276 (b), phosphorylated p65 Ser-281 (c), phosphorylated p65 Ser-311 (d), phosphorylated p65 Ser-468 (e), phosphorylated p65 Ser-529 (f), phosphorylated p65 Ser-536 (g), and phosphorylated p65 Thr-435 (h) were detected by Western blot. Data shown are representative of 5 independent experiments in duplicate and are expressed as mean ± SD. ^∗^*P* < 0.05 versus NG and ^#^*P* < 0.05 versus HG.

The human Müller cell line, MIO-M1, indicated similar trends as observed in HREC where not all sites resulted in increased phosphorylation with high glucose exposure. As shown in Figure 3, p65 subunit sites Thr-254 (*A*), Ser-281 (*C*), Ser-311 (*D*), Ser-468 (*E*), and Thr-435 (*H*) were activated due to high glucose alone, while no differences in phosphorylation were observed for Ser-276 (*B*), Ser-529 (*F*), and Ser-536 (*G*) compared to normal-glucose controls. However, both Ser-529 and Ser-536 sites did reveal significantly increased phosphorylation after treatment with IL-1*β* and TNF-*α*. Unlike HREC though, IL-1*β* and TNF-*α* treatment did not appear to have as strong of an effect on Müller cells; enhanced phosphorylation beyond high glucose-induced effects was limited to Ser-468, Thr-254 (IL-1*β* only at 24 h), and Ser-311 (TNF-*α* only at 10 and 30 min). IL-4 treatment, however, significantly downregulated phosphorylation of NF-*κ*B p65 at Thr-254, Ser-276, Ser-281, Ser-468, and Thr-435 compared to high glucose. In addition, although phosphorylation levels of Ser-276 did not change in response to high-glucose or proinflammatory cytokine treatment, IL-4 downregulated phosphorylation beyond normal glucose controls. Results of phosphorylation on different sites with high-glucoseversus cytokine treatment are summarized in Table 2.

### 3.3. Effects of High Glucose and Cytokine Treatment on Transcription of Downstream Genes Associated with NF-*κ*B Activation in HREC and Müller Cells

**Figure 4:**
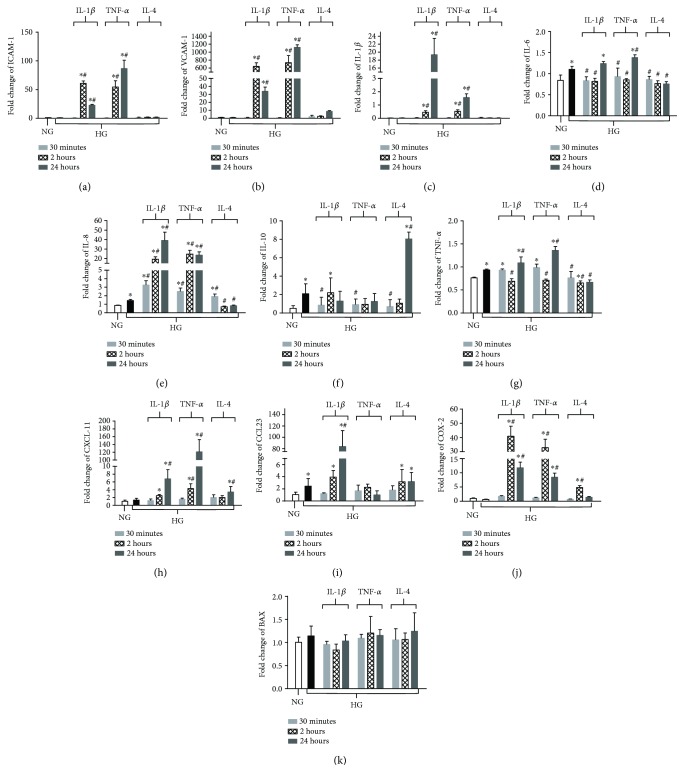
mRNA expression levels of NF-*κ*B regulated inflammatory genes after high glucose exposure and pro- versus anti-inflammatory cytokine treatment in HREC. Cells were cultured under normal-glucose (NG, 5 mM) and high-glucose (HG, 25 mM) conditions followed by TNF-*α* and IL-1*β* versus IL-4 treatment. mRNA levels of ICAM-1 (a), VCAM-1 (b), IL-1*β* (c), IL-6 (d), IL-8 (e), IL-10 (f), TNF-*α* (g), CXCL-11 (h), CCL-23 (i), COX-2 (j), and BAX (k) were detected by real-time RT-PCR over time. Data shown are representative of 5 independent experiments in duplicate and are expressed as mean ± SD. ^∗^*P* < 0.05 versus NG and ^#^*P* < 0.05 versus HG.

Transcription of selected genes known to be regulated by NF-*κ*B as well as pathogenic in DR was assessed after high glucose exposure and cytokine treatment in both HREC and Müller cells. Analysis of gene expression in HREC (Figure 4) indicated that high glucose upregulated the transcript levels of interleukin-6 (IL-6) (*D*), IL-8 (*E*), IL-10 (*F*), TNF-*α* (*G*), and C-C motif chemokine ligand 23 (CCL23) (*I*). High glucose had no effect on the expression of ICAM-1 (*A*), VCAM-1 (*B*), IL-1*β* (*C*), C-X-C motif chemokine 11 (CXCL11) (*H*), cyclooxygenase-2 (COX-2) (*J*), or bcl-2 associated X protein (BAX) (*K*). When high glucose exposure was combined with IL-1*β* treatment, transcription levels of ICAM-1, VCAM-1, IL-1*β*, IL-6 (versus normal glucose only), IL-8, TNF-*α*, CXCL11, CCL23, and COX-2 were significantly upregulated over both normal glucose and high glucose alone. mRNA levels of IL-10 were lower than high glucose alone at 30 min posttreatment with IL-1*β*, while BAX did not change from basal expression levels. Treatment with TNF-*α* revealed similar trends compared to IL-1*β* and resulted in upregulation of ICAM-1, VCAM-1, IL-1*β*, IL-6, IL-8, TNF-*α*, CXCL11, and COX-2. In contrast, IL-10 expression was decreased compared to high glucose only at 30 min and no differences were detected regarding CCL23 or BAX compared to basal expression. Despite high glucose exposure, IL-4 treatment decreased the mRNA levels for IL-6, IL-8, and TNF-*α*. No differences were observed in ICAM-1, VCAM-1, IL-1*β*, or BAX when compared to normal-glucose controls. In addition, IL-4 treatment upregulated IL-10 and CXCL11 at 24 h and COX-2 at 2 h time points compared to high glucose only. High glucose-induced upregulation of CCL23 mRNA remained unchanged after IL-4 treatment.

**Figure 5:**
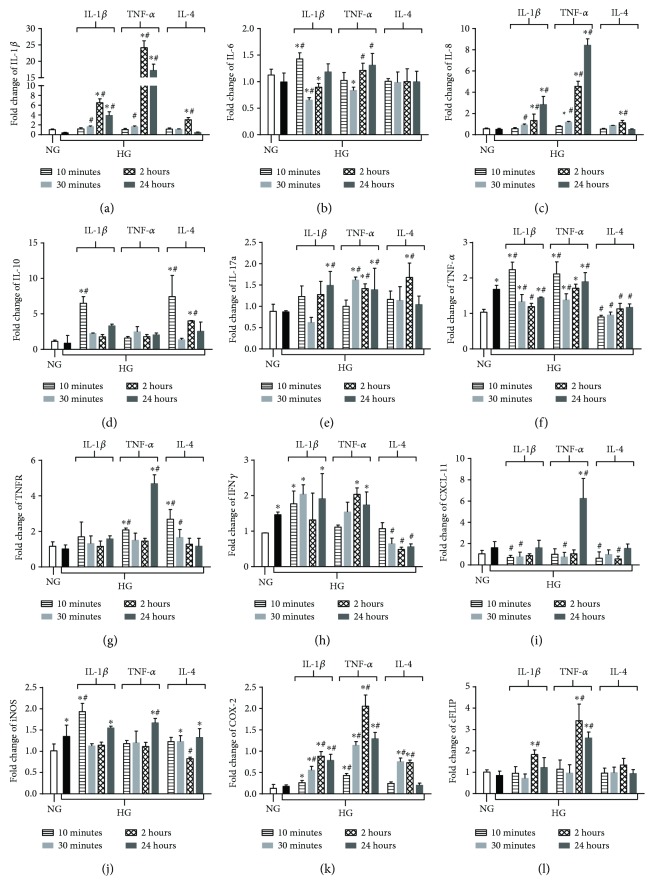
mRNA expression of NF-*κ*B-regulated inflammatory genes under high-glucose conditions and cytokine polarization in Müller cells. Müller cells were cultured under normal-glucose (NG, 5 mM) and high-glucose (HG, 25 mM) conditions followed by TNF-*α* and IL-1*β* versus IL-4 treatment over time. mRNA levels of IL-1*β* (a), IL-6 (b), IL-8 (c), IL-10 (d), IL-17a (e), TNF-*α* (f), TNFR (g), IFN*γ* (h), CXCL-11 (i), iNOS (j), COX-2 (k), and cFLIP (l) were detected by real-time RT-PCR. Data shown are representative of 5 independent experiments in duplicate and are expressed as mean ± SD. ^∗^*P* < 0.05 versus NG and ^#^*P* < 0.05 versus HG.

In Müller cells, high glucose exposure resulted in upregulation of mRNA transcripts for a limited number of mediators (Figure 5): TNF-*α* (*F*), interferon-gamma (IFN*γ*) (*H*), and inducible nitric oxide synthase (iNOS) (*J*). Addition of proinflammatory IL-1*β* or TNF-*α* under high-glucose conditions increased the mRNA levels for IL-1*β* (*A*), IL-6 (*B*), IL-8 (*C*), interleukin-17A (IL-17A) (*E*), TNF-*α* (*F*), TNFR (TNF-*α* treatment only) (*G*), iNOS (*J*), COX-2 (*K*), and cellular FLICE inhibitory protein (cFLIP) (*L*). Similar to HREC, CXCL11 (*I*) was exclusively upregulated by TNF-*α*, not IL-1*β*, in Müller cells. IL-4 upregulated the mRNA expression of IL-10 (*D*) and COX-2, yet decreased the expression of TNF-*α*, IFN*γ*, CXCL11, and iNOS. No effect was observed regarding IL-6 or cFLIP expression after IL-4 treatment.

### 3.4. Protein Analysis of NF-*κ*B-Regulated Genes in HREC and Müller Cells after High Glucose Exposure and Cytokine Treatment

**Figure 6:**
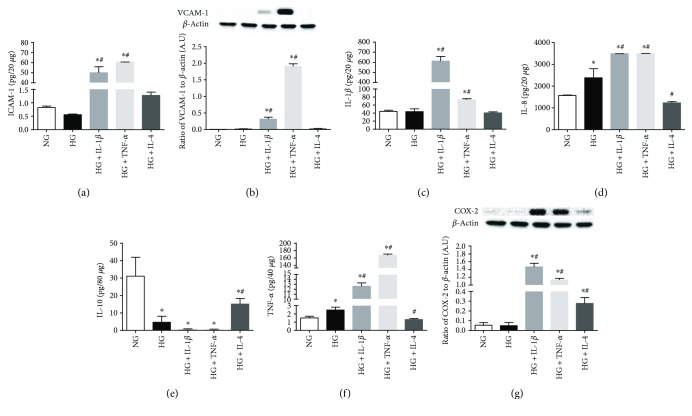
Protein levels of select NF-*κ*B-regulated inflammatory mediators in HREC after high glucose exposure and pro- versus anti-inflammatory cytokine polarization. HRECs were cultured under normal-glucose (NG, 5 mM) and high-glucose (HG, 25 mM) conditions followed by TNF-*α* and IL-1*β* versus IL-4 treatment for 24 hours. Protein levels of ICAM-1 (a), VCAM-1 (b), IL-1*β* (c), IL-8 (d), IL-10 (e), TNF-*α* (f), and COX-2 (g) were detected by ELISA or Western blot. Data shown are representative of 5 independent experiments in duplicate and are expressed as mean ± SD. ^∗^*P* < 0.05 versus NG and ^#^*P* < 0.05 versus HG.

Based on the mRNA expression, several genes were selected to further analyze protein levels after 24 h of high glucose exposure and pro-/anti-inflammatory cytokine treatments (Figure 6). Consistent with mRNA results, protein levels of ICAM-1 (*A*), VCAM-1 (*B*), IL-1*β* (*C*), IL-8 (*D*), TNF-*α* (*F*), and COX-2 (*G*) were similar in high and normal glucose. IL-10 (*E*), which was downregulated, was the only molecule that did not show corresponding mRNA expression and protein levels after high glucose exposure. When HRECs were exposed to high glucose and proinflammatory cytokine IL-1*β* or TNF-*α*, protein levels were significantly elevated over both normal and high glucose for all molecules (except IL-10)—including ICAM-1, VCAM-1, IL-1*β*, IL-8, TNF-*α*, and COX-2. As mentioned, IL-10 was significantly reduced in the presence of either proinflammatory cytokine compared to normal glucose controls. On the other hand, IL-4 treatment abrogated high glucose-induced changes in IL-8, TNF-*α*, and upregulated IL-10. Further, IL-4 treatment had no effect on ICAM-1, VCAM-1, or IL-1*β*, which remained similar to basal levels observed in normal glucose. Similar to mRNA expression, COX-2 protein levels were significantly elevated with IL-4 treatment, but not as elevated after IL-1*β* or TNF-*α* treatments.

**Figure 7:**
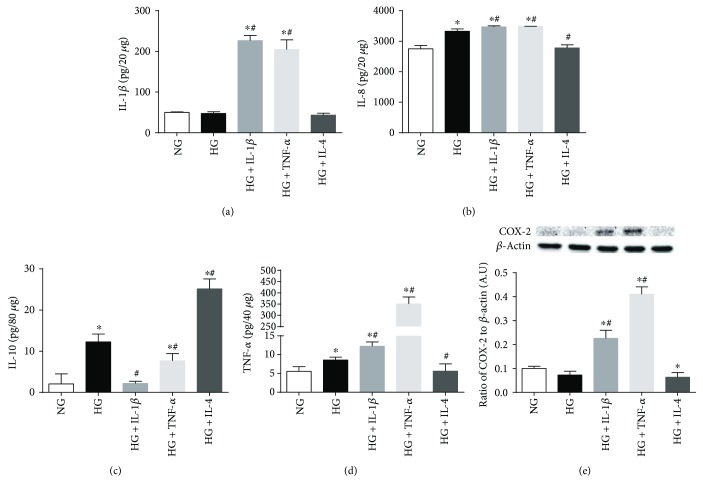
Protein levels of select NF-*κ*B-regulated inflammatory mediators in Müller cells exposed to high glucose with cytokine treatments. Müller cells were cultured under normal-glucose (NG, 5 mM) and high-glucose (HG, 25 mM) conditions followed by TNF-*α* and IL-1*β* versus IL-4 treatment for 24 hours. Protein levels of IL-1*β* (a), IL-8 (b), IL-10 (c), TNF-*α* (d), and COX-2 (e) were detected by ELISA or Western blot. Data shown are representative of 5 independent experiments in duplicate and are expressed as mean ± SD. ^∗^*P* < 0.05 versus NG and ^#^*P* < 0.05 versus HG.

Protein levels were also examined at 24 h in Müller cells exposed to high glucose and cytokine treatments, as shown in Figure 7 for IL-1*β* (*A*), IL-8 (*B*), IL-10 (*C*), TNF-*α* (*D*), and COX-2 (*E*). Exposure to high glucose had no effect on IL-1*β* and COX-2 when compared to basal levels observed in normal glucose. However, IL-8, TNF-*α*, and surprisingly IL-10 were significantly increased with high glucose. As expected, treatment with IL-1*β* and TNF-*α* resulted in significant increases of IL-1*β*, IL-8, TNF-*α*, and COX-2 over both normal and high glucose. IL-10, on the other hand, was significantly decreased compared to high glucose. These effects were consistently reversed following IL-4 treatment—reducing IL-8 and TNF-*α* to basal levels observed with normal-glucose exposure. IL-10, however, was further enhanced after IL-4 treatment over high glucose.

## 4. Discussion

Phosphorylation is one of the prerequisite steps in NF-*κ*B p65 activation. It has been found to be highly important for recruitment of transcription factors and subsequent binding of the p65 subunit with its target genes, as demonstrated by various NF-*κ*B p65 site-specific studies [33–45]. Although it has been shown over a decade ago that high glucose activates NF-*κ*B in pericytes [46] and vascular smooth muscle cells [47], more recent reports have indicated that NF-*κ*B activation in RECs is rather due to paracrine influences from other retinal cells such as Müller cells and pigment epithelial cells [15]. In the current study, we demonstrate that high glucose induces differential phosphorylation of NF-*κ*B in two retinal cell types, HREC and Müller cells. Similar trends were observed regarding high glucose-induced phosphorylation of three p65 subunit sites—Thr-254, Thr-435, and Ser-468—in both HREC and Müller cells. High glucose-induced phosphorylation of Ser-276 and Ser-529 was specific to HREC, however, while phosphorylation of Ser-281 and Ser-311 was restricted to Müller cells. Ser-536, on the other hand, was not influenced by high glucose exposure in either cell type. These findings are important given that many of the studies looking at hyperglycemia-induced NF-*κ*B activation focus on Ser-536, which may be reflecting the effects of other mediators instead.

Phosphorylation of Ser-311 by *ζ*PKC has been associated with IL-6 transcription [37]. Both Ser-276 and Ser-311 phosphorylation has been found to promote interaction with CREB, enhancing the NF-*κ*B response [34, 37]. Ser-536 phosphorylation defines separate pathways of NF-*κ*B activation of canonical and noncanonical patterns, activating transcription of IL-8 and ICAM-1, both of which are important for leukostasis activation [42]. Mutation of Ser-536 prevents CBP recruitment [41]. Phosphorylation of Ser-529 and Ser-536 has been shown to be associated with translocation of NF-*κ*B subunits [36, 39]. In the current study, we showed that Ser-311 and Ser-536 were not responsive to high glucose exposure in HREC. Similarly, Ser-529 and Ser-536 remained unchanged in Müller cells. Yet these three sites were significantly activated by inflammatory cytokines, as discussed next.

Beyond high glucose-induced effects, we also characterized two major proinflammatory cytokines, IL-1*β* and TNF-*α*, which are potent activators of NF-*κ*B and produced early in the development of DR [48–50]. Both cytokines were shown to be potent activators of all p65 subunit sites, indicating an important role for Ser-536 in particular, regarding the canonical NF-*κ*B pathway. Though we demonstrated that select p65 sites were differentially influenced by high glucose exposure in HREC and Müller cells, the presence of proinflammatory cytokines (IL-1*β* and TNF-*α*) had a robust effect on all p65 phosphorylation sites tested.

The complex nature of DR pathogenesis includes a wide spectrum of mediators, such as advanced glycation end products (AGEs), ROS, protein kinase C (PKC) pathway, polyol pathway, and inflammatory pathways [51]. Upon activation by any of these pathogenic molecules related to the development of DR, NF-*κ*B induces further transcription of various inflammatory mediators that exacerbate the disease state. Such inflammatory mediators include IL-1*β*, TNF-*α*, IL-6, IL-8, and monocyte chemotactic protein-1 (MCP-1) [52–56]; iNOS [57]; COX-2 [58]; adhesion molecules ICAM-1 and VCAM-1 [59]; and apoptotic molecules, Fas and Fas ligand [60]. High glucose exposure resulted in an upregulation of IL-8 and TNF-*α* protein levels, which suggests that hyperglycemic conditions alone are somewhat limited in inducing an inflammatory response in either HREC or Müller cells. However, the fact that TNF-*α* was upregulated after high glucose exposure in both cell types underlines the detrimental effects of diabetes. As a major contributor to the pathogenesis of DR, TNF-*α* has been shown to mediate inflammation [61], regulate the breakdown of the blood-retinal barrier [62], and direct apoptosis of retinal residential cells [49]. As evidenced from proinflammatory cytokine treatments, once TNF-*α*/IL-1*β* is present in the microenvironment, the cascade of inflammatory events is exacerbated, which potentiates a state of chronic inflammation within the retina. This is supported by results indicating that TNF-*α* and IL-1*β* resulted in significant upregulation of downstream inflammatory mediators compared to high glucose alone. These findings agree with the trends observed from our NF-*κ*B p65 phosphorylation results, further indicating the important role for proinflammatory cytokines in the early pathogenesis of DR, as high glucose appears to have a markedly reduced effect comparatively. Although the current study did not look at the effect of proinflammatory cytokines under normal-glucose conditions, others have shown that IL-1*β* induces IL-6 expression in human Müller cells through NF-*κ*B activation [56] and that IL-1*β* accelerates apoptosis of retinal endothelial cells [48].

The current study is the first, to our knowledge, to demonstrate the suppressive effect of IL-4 on the phosphorylation of NF-*κ*B p65. Although it has been reported that IL-4 induces NF-*κ*B p52 [63], there is no established evidence regarding IL-4's effect on NF-*κ*B p65. The fact that IL-4 suppressed phosphorylation in 4 of 8 p65 sites in HREC and 5 of 8 sites in Müller cells under high-glucose conditions demonstrates its protective effect, resulting in reduced IL-6, IL-8, and TNF-*α* (HREC) and TNF-*α*, IFN*γ*, CXCL-11, and iNOS (Müller cells). IL-4 treatment also upregulated IL-10, which has been shown to inhibit IL-8 [64] and regulate TNF-*α* signaling [65]. In addition, the fact that IL-4 promoted expression of COX-2 in both cell types may be associated with COX-2 derived prostanoids, which are important at low levels in the development of a “healthy” immune response. IL-4 also enhanced I*κ*B*α* levels in Müller cells, which may contribute to its inhibition of NF-*κ*B activation. Although further study is needed to link the different p65 phosphorylation sites to the respective activation of downstream inflammatory mediators, that IL-4 not only inhibited phosphorylation of multiple p65 sites but also suppressed NF-*κ*B-induced mediators provides an attractive avenue for treatment against the pathogenesis of DR. Given that obesity is associated not only with prediabetes but also with a state of chronic inflammation [66], addressing the detrimental effects of inflammatory mediators earlier could delay the progression of DR more effectively than once a patient is considered diabetic.

I*κ*B*α* inhibits NF-*κ*B by sequestering p65 in the cytoplasm. When phosphorylated, ubiquitinated, and then degradated, I*κ*B*α* releases NF-*κ*B p65 so that it can dimerize with p50 then translocate into the nucleus to trigger activation of the downstream canonical NF-*κ*B pathway [67]. Beyond the traditional view of I*κ*B*α*, recent reports have pointed out the existence of free I*κ*B*α* in the cytoplasm as an intrinsic unstable molecule, which is important for activation of NF-*κ*B itself and independent of IKK phosphorylation and ubiquitination [68]. This adds to the complexity of the NF-*κ*B pathway and promotes the possibility of I*κ*B*α* involvement not only in the alternative NF-*κ*B pathway but also in an IKK kinase and p65-p50-independent manner. Although we showed that proinflammatory cytokines significantly upregulate I*κ*B*α* degradation in both cell types, high glucose alone resulted in decreased I*κ*B*α* levels in Müller cells.

## 5. Conclusions

In summary, high glucose-induced site-specific differential phosphorylation of NF-*κ*B p65 in HREC and Müller cells was characterized in the presence of pro- and anti-inflammatory cytokines, demonstrating the importance of inflammatory mediators in exacerbating the retinal response. In addition, IL-4 was shown to inhibit high glucose-induced NF-*κ*B phosphorylation at multiple p65 sites in HREC and Müller cells, resulting in decreased NF-*κ*B activation. The current study is the first to characterize high glucose-induced NF-*κ*B p65 phosphorylation and reveal IL-4's regulatory effect on this activity. As such, it underscores the importance of recognizing the differential influence of hyperglycemic conditions between multiple retinal cell types and that the observed effects go beyond a single phosphorylation site on the NF-*κ*B p65 subunit. These findings are important in understanding the pathologic events associated with DR. NF-*κ*B activation is a major event associated with high glucose-induced changes in the retina; however, we can conclude from this study that understanding how NF-*κ*B is activated in each cell type is relevant since cell types and phosphorylation sites are differentially responsive to both high glucose and inflammatory mediators.

## Figures and Tables

**Table 1:** Primer sequences.

Genes	Definition	Forward (5′ to 3′)	Reverse (5′ to 3′)
ICAM-1	Intercellular adhesion molecule 1	CTTCGTGTCCTGTATGGCCC	CACATTGGAGTCTGCTGGGA
VCAM-1	Vascular cell adhesion protein 1	GTCAATGTTGCCCCCAGAGATA	ACAGGATTTTCGGAGCAGGA
IL-1*β*	Interleukin-1 beta	AGAAGTACCTGAGCTCGCCA	CTGGAAGGAGCACTTCATCTGT
IL-6	Interleukin-6	TACAGGGAGAGGGAGCGATA	CTCAGACATCTCCAGTCCTCT
IL-8	Interleukin-8	AGAGCCAGGAAGAAACCACC	GGCAAAACTGCACCTTCACAC
IL-10	Interleukin-10	AAGACCCAGACATCAAGGCG	AATCGATGACAGCGCCGTAG
TNF-*α*	Tumor necrosis factor alpha	AGGCGCTCCCCAAGAAGACA	TCCTTGGCAAAACTGCACCT
TNFR	Tumor necrosis factor receptor	CCAGTGCGTTGGACAGAAGG	GAAGAATCTGAGCTCCCGGTG
CXCL-11	C-X-C motif chemokine 11	TTGTTCAAGGCTTCCCCATGT	CCACTTTCACTGCTTTTACCCC
CCL-23	C-C motif chemokine ligand 23	CTGGACATGCTCTGGAGGAGA	GGAGTGAACACGGGATGCTT
COX-2	Cyclooxygenase 2	GCTGTTCCCACCCATGTCAA	AAATTCCGGTGTTGAGCAGT
BAX	Bcl-2-associated X protein	CATGGGCTGGACATTGGACT	GGCAGCCCCCAACCAC
IFN*γ*	Interferon gamma	TGGAAAGAGGAGAGTGACAGA	ACACTCTTTTGGATGCTCTGGT
IL-17a	Interleukin-17A	CCTTGGAATCTCCACCGCAA	GTGGTAGTCCACGTTCCCAT
iNOS	Inducible nitric oxide synthase	GGACCCTGCAGACAGGC	TTCTTCACTGTGGGGCAAGG
cFLIP	Cellular FLICE-inhibitory protein	CAGCAGGTCTGAGCTTGTCC	AGTGGGGGAGTTGCCCG

**Table 2:** Summarization of NF-*κ*B p65 phosphorylation sites.

NF-*κ*B p65 phosphorylation sites	Main functions	HREC			Müller cells		
		HG	HG + IL-1*β* or TNF-*α*	HG + IL-4	HG	HG + IL-1*β* or TNF-*α*	HG + IL-4
Thr-254	p65 transactivation [69]	↑	↑↑↑	↓	↑↑↑	↑↑↑	↓↓
Ser-276	CBP recruitment, system inflammation [33, 44]; Translocation of p65 [43]	↑↑↑	↑↑↑	None	None	None	↓↓↓
Ser-281	Translocation of p65 [43]	None	↑↑	↓	↑↑↑	↑↑↑	↓↓↓
Ser-311	IL-6 transcription and CBP recruitment [37]	None	↑↑↑	↓	↑	↑↑↑	None
Ser-468	ICAM-1, VCAM-1 transcription [45]	↑↑↑	↑↑↑	None	↑	↑↑↑	↓
Ser-529	Translocation of p65 [36]	↑	↑↑↑	None	None	↑↑↑	None
Ser-536	ICAM-1, IL-8 transcription [42]; CBP recruitment [41]; translocation of p65 [39]	None	↑↑↑	None	None	↑↑↑	↑
Thr-435	p65 transactivation [70]	↑↑↑	↑↑↑	↓	↑↑	↑↑↑	↓↓↓

↑/↓, ↑↑/↓↓, and ↑↑↑/↓↓↓ indicate *P* < 0.05, 0.01, and 0.0001, respectively, versus normal-glucose/high-glucose controls. None indicates no changes were observed. Results were derived from the peak change observed among the different time points examined.

## Abbreviations

DM: Diabetic mellitus
DR: Diabetic retinopathy
VEGF: Vascular endothelial growth factor
REC: Retinal endothelial cell
ROS: Reactive oxygen species
IL-1R: IL-1 receptor
TNFR: TNF receptor
PRRs: Pattern recognition receptors
CREB: cAMP-response element binding protein
CBP: CREB binding protein
HAT: Histone acetyltransferase
IL-1*β*: Interleukin-1 beta
TNF-*α*: Tumor necrosis factor-alpha
IL-4: Interleukin-4
PMN: Polymorphonuclear leukocytes
HREC: Human retinal microvascular endothelial cells
DMEM: Dulbecco's modified Eagle medium
FBS: Fetal bovine serum
VCAM-1: Vascular cell adhesion protein 1
ICAM-1: Intercellular adhesion molecule 1
IL-8: Interleukin-8
IL-10: Interleukin-10
IL-6: Interleukin-6
CCL23: C-C motif chemokine ligand 23
CXCL-11: C-X-C motif chemokine 11
COX-2: Cyclooxygenase 2
BAX: Bcl-2-associated X protein
IFN*γ*: Interferon gamma
iNOS: Inducible nitric oxide synthase
IL-17A: Interleukin-17A
cFLIP: Cellular FLICE inhibitory protein
AGEs: Advanced glycation end products
PKC: Protein kinase C
MCP-1: Monocyte chemotactic protein-1.
